# Barriers and facilitators of appropriate antibiotic use in primary care institutions after an antibiotic quality improvement program – a nested qualitative study

**DOI:** 10.1186/s12877-022-03161-w

**Published:** 2022-05-27

**Authors:** Nicolay Jonassen Harbin, Morten Lindbæk, Maria Romøren

**Affiliations:** 1 Antibiotic Center for Primary Care, Department of General Practice, Institute of Health and Society, University of Oslo, Postboks 1130 Blindern, 0317 Oslo, Norway; 2 Centre for Medical Ethics, Institute of Health and Society, Faculty of Medicine, University of Oslo, Oslo, Norway

**Keywords:** Nursing home, Municipal acute care unit, Antibiotic stewardship program, Barriers, Facilitators, Urinary tract infection, Life-prolonging treatment

## Abstract

**Background:**

Antibiotic prescribing by physicians in primary care institutions is common and affected by several factors. Diagnosis and treatment of infections in a nursing home (NH) resident is challenging, with the risk of both under- and overtreatment. Identifying barriers and facilitators of appropriate antibiotic prescribing in NHs and municipal acute care units (MACUs) is essential to ensure the most adequate antibiotic treatment possible and develop future antibiotic stewardship programs.

**Methods:**

After implementing a one-year antibiotic quality improvement program, we conducted six semi-structured focus group interviews with physicians (*n* = 11) and nurses (*n* = 14) in 10 NHs and 3 MACUs located in the county of Østfold, Norway. We used a semi-structured interview guide covering multiple areas influencing antibiotic use to identify persistent barriers and facilitators of appropriate antibiotic prescribing after the intervention. The interviews were audio-recorded and transcribed verbatim. The content analysis was performed following the six phases of thematic analysis developed by Braun and Clarke.

**Results:**

We identified thirteen themes containing barriers and facilitators of the appropriateness of antibiotic use in primary care institutions. The themes were grouped into four main levels: Barriers and facilitators 1) at the clinical level, 2) at the resident level, 3) at the next of kin level, and 4) at the organisational level. Unclear clinical presentation of symptoms and lack of diagnostic possibilities were described as essential barriers to appropriate antibiotic use. At the same time, increased availability of the permanent nursing home physician and early and frequent dialogue with the residents’ next of kin were emphasized as facilitators of appropriate antibiotic use. The influence of nurses in the decision-making process regarding infection diagnostics and treatment was by both professions described as profound.

**Conclusions:**

Our qualitative study identified four main levels containing several barriers and facilitators of appropriate antibiotic prescribing in Norwegian NHs and MACUs. Diagnostic uncertainty, frequent dialogue with next of kin and organisational factors should be targeted in future antibiotic stewardship programs in primary care institutions. In addition, for such programs to be as effective as possible, nurses should be included on equal terms with physicians.

## Background

Antimicrobial resistance is an increasing challenge worldwide [1], and the call for prudent use of antibiotics is in demand to slow down and reverse further resistance development [2]. Previous studies have shown that 1/2 to more than 3/4 of nursing home (NH) residents receive one or more courses of antibiotics during a calendar year [3–6]. Bacterial infections requiring antimicrobial treatment has a high prevalence in long-term care residents compared to elderly living at home [7], many of them deemed inappropriate [8–10].

Prescribing antibiotics can be classified either as medically or ethically appropriate based on the circumstances of the specific antibiotic-requiring infection. Medically appropriate antibiotic use typically covers the clinical, microbiological, pharmacokinetic and dynamic aspects of the specific cases. Simultaneously, antibiotic treatment among old and frail NH residents is primarily about prolonging life, which raises the question of whether antibiotic treatment is appropriate or inappropriate from an ethical perspective. Ethical questions make infection diagnostics and treatment a more significant challenge for NH physicians and nurses, as multiple comorbidities, polypharmacy, speech and hearing disabilities, cognitive debilitation, and atypical symptom manifestation of infections is more common in the NH population [11–15]. Limited diagnostic on-site possibilities at the NHs further complicate diagnosis and treatment of infections [16]. Next of kin is usually more involved in promoting NH residents’ interests and demands than in their earlier adult life. Although the Norwegian health law demands more next of kin involvement when residents cannot consent, the providing physician has to make final decisions in cases involving consent incompetence [17]. Besides apparent advantages and benefits of next of kin involvement, this guardianship role may lead to tension, disagreement and conflict between relatives and health care professionals regarding infection treatment [18, 19].

Although the final decision to prescribe antibiotics or not is taken by one or several medical physicians, the decision-making of prescribing is multifactorial and complex. Previous studies have identified several factors influencing antibiotic prescribing in hospitals and general practice, for example, physician-specific and patient-related factors, availability of diagnostic tools and local antibiotic resistance data, patient satisfaction and cultural and organizational factors [20–31]. Many of the factors identified in general practice and hospitals may apply for NHs, and some issues are specific to NHs affecting antibiotic prescribing. Studies on antibiotic prescribing decisions in NHs and older aged patients have identified the availability of evidence-based guidelines, physicians’ habits, perceived risks of antibiotic prescribing, the influence of other health care professionals, and residents’ current clinical situation and medical history as important factors influencing antibiotic treatment duration and the decision-making whether or not to prescribe antibiotics [16, 32–36].

Responding to the emerging antimicrobial resistance threat, the Norwegian Government published its “National Action Plan against Antibiotic Resistance in the Health Services” in 2016 [37]. As part of the plan, the Antibiotic Centre for Primary Care launched the “RASK” intervention in the county of Østfold, a quality improvement programme aiming to optimize treatment of infections and improve antibiotic prescribing in Norwegian NHs and municipal acute care units (MACUs) [38]. The intervention aimed to increase the knowledge regarding appropriate antibiotic treatment and increase the awareness of the institutions’ antibiotic prescribing patterns.

Previous Norwegian studies have found a wide variation in total antibiotic use between Norwegian NHs, indicating a potential for improving antibiotic prescribing in this sector [39, 40]. In addition, we have identified only one previous Norwegian qualitative study on factors influencing antibiotic treatment in NH residents, who primarily investigated the ethical problems related to intravenous antibiotic administration perceived by NH nurses [18]. Further studies, investigating the current topic on a broader level is warranted. Therefore, this focus group study aimed to in-depth explore both physicians’ and nurses’ perceptions of persisting barriers and facilitators of appropriate antibiotic use in Norwegian NHs and MACUs after the implementation of a structured antibiotic improvement program.

## Methods

This article conforms to the *“*Standards for Reporting Qualitative Research (SRQR): 21-items checklist” [41].

### Study setting

The current study was initiated after completing the “RASK” intervention in Østfold county, located in the South-Eastern part of Norway, which lasted from October 2016 to October 2017. In Norway, NHs may be classified as long-term, short-term or mixed (both long- and short-term) based on the residency. In addition to ordinary NHs, we have municipal acute care units (MACUs) to alleviate the use of hospital services. Although classified as a part of the NH sector, MACUs differ from traditional NHs in that the patients live at home and are admitted due to acute onset disease by general practitioners during regular working hours and out-of-hours. When the patients have been treated, they are usually discharged home.

We invited all 37 NHs and MACUs in the county to the intervention, resulting in 34 institutions participating in the project. NH and MACU physicians, nurses and other healthcare professionals from included institutions were then invited to a one-day conference with professional presentations and workshops on infections and appropriate use of antibiotics. All participating institutions received a report presenting their antibiotic use based on sales statistics from supplying pharmacies, compared to other participating institutions in the county. After the starting conference, participants were instructed to arrange educational activities on the same topics for their colleagues and set a goal for their institution during the one-year project period. In addition, the institutions were asked to register bi-monthly point prevalence surveys on antibiotic use and indication, and tailor-made clinical checklists were offered as tools to be used during the intervention year. Follow-up conferences were held after six and 12 months, and participating institutions received new antibiotic reports for further academic audit and feedback. All physicians and nurses working in the NHs or MACUs that took part in the intervention were invited orally at the final “RASK” conference to participate in the current study, and in addition they were personally invited by email or telephone. Willing informants were included until we decided that a saturation point had been reached. The saturation point was assessed continuously by comparing the current interview with summaries and transcripts of previous interviews, and decided upon when no new themes and no additional information on pre-existing themes occurred. We conducted six focus group interviews between October 2017 and December 2018 with 11 physicians and 14 nurses from 13 institutions. The participants did not work in the same institution, except from one interview where one physician and three nurses were employed at the same MACU ward. Nine physicians and six nurses had participated at one or more conferences during the intervention year, and all participants were familiar with the programme through the antibiotic reports, educational material, and the use of intervention tools at the institutions. Each focus group consisted of three to six participants, a size range decided upon to obtain interactive group discussions during the interviews and to increase the involvement level for each participant. Four of the interviews were conducted with both physicians and nurses mixed to explore the dynamics between the two occupational groups. The two last interviews were conducted with only physicians in one interview and only nurses in the other to see if we received other information when the groups were interviewed separately.

### Researcher characteristics

NJH is a part time NH physician working at a short-term NH in one of the municipalities that participated in the “RASK” intervention. In addition, he was the responsible coordinator for the “RASK” intervention in the county of Østfold. NJH had no experience in qualitative research prior to conducting the current study. ML is a long-time GP and a researcher in the field of antibiotic prescribing in general practice and NHs, and had the overall leadership responsibility for the “RASK” intervention in Østfold. MR is a GP and a long-time researcher in the field of NH medicine and general practice. Both ML and MR have extensive prior experience and training in conducting qualitative research. All authors share a common interest in quality improvement in primary care institutions, and factors affecting antibiotic prescribing in particular. Based on the authors background, one could imagine that the informants would formulate their answers based on what they thought was expected to be answered. However, we perceived the discussions in the interviews as open and rich, and that the health personnel talked uncensored about their thoughts, experiences and dilemmas in their clinical work. The prior knowledge of the organizational structure and clinical everyday life rather was an advantage in penetrating and understanding the informants’ stories and perceptions.

### Data collection

The interview duration varied from 56 minutes to 87 minutes, with a mean overall duration of 75 minutes. NJH was the main interviewer in all six interviews, while ML and MR participated as co-interviewers in one and five interviews, respectively. We used a semi-structured interview guide that NJH, MR, and ML developed. The interview guide contained four main topics; 1) factors influencing physicians’ antibiotic prescribing, 2) factors influencing physicians’ choice to deviate from antibiotic guidelines, 3) influence of nurses on physicians’ antibiotic prescribing and 4) what ethical dilemmas physicians and nurses experience regarding antibiotic treatment. The informants were provided with written information about the main topics of the study by email in advance. All interviews were started by shortly describing the main topics, followed by encouraging the informants to describe two experienced cases where antibiotic treatment had been initiated; one where there had been no doubt and the other where there had been hesitations regarding the treatment. The main topics were then presented to the informants step-wise through the interviews to initiate discussions in the group. The informants were moderated if they deviated greatly from the topics. When saturation on the relevant topics became evident, the interviewers moved on to the next topic in the interview guide. The interviewers also engaged informants who were not as involved in the discussions and complemented with in-depth questions along the way as needed.

### Data analysis

The interviewers discussed the interviews immediately after completion of each interview, and a summary for each interview was written and discussed further by email. All interviews were audio-recorded and transcribed verbatim. We based the analyses on the six phases of thematic analysis developed by Braun and Clarke [42], primarily with a inductive and semantic approach: 1) familiarizing with the depth and breadth of the content by reading repeatedly through the interviews to gain a general impression, 2) generating initial codes for the entire material using both theory and data-driven approaches, 3) searching for themes and re-sorting the initial codes into potential themes, 4) reviewing potential themes at the level of the coded data extracts and creating a candidate thematic map and secondly considering the validity of individual themes and the candidate thematic map in relation to the data set, 5) further defining, refining and naming the themes and identifying barriers and facilitators of appropriate antibiotic use, 6) identifying, defining and naming overarching levels, and further group the themes at the appropriate level, 7) production of the article including illustrative extracts from the material that captures the essence of the points demonstrated. NJH transcribed the interviews and performed the initial coding and analysis of the content. MR and ML further evaluated the transcribed data material, as well as the initial coding of the content. All authors participated substantially in the process of searching, defining, reviewing and naming relevant themes and in the article production. The qualitative data analysis software program NVIVO 12 was used for analyses and data management [43].

### Ethical approval

All participants provided written consent prior to the interviews. We replaced any names and places with numbers and characters in the transcribed text to protect the anonymity of the participants. The Regional Committees for Medical and Health Research Ethics of South-East Norway granted ethics approval for the study (ref.: 2017/1711), and the Norwegian Centre for Research Data approved data protection (55,887 / 3 / LAR).

## Results

The demographic characteristics of the informants are presented in Table 1.

**Table 1:** Characteristics of the study informants

Demographics		Physicians (*n =* 11)	Nurses (*n =* 14)	Overall (*n =* 25)
Sex	Female	7	13	20
	Male	4	1	5
Age	Mean (range)	42 (32 – 64)	43 (25 – 62)	43 (25 – 64)
Years clinical experience	Mean (range)	14 (4 – 37)	16 (2 – 41)	15 (2-41)
Type of facility	Nursing home	7	10	17
	Municipal acute care unit	4	4	8
Speciality	Nursing home	General practitioner (1)	Geriatric and palliative medicine (1)	–
		Internal medicine (2)	Rehabilitation medicine (1)	
		In specialisation (3)	Registered nurse (8)	
		No specialisation (1)		
	Municipal acute care unit	General practitioner (1)	Acute geriatric medicine (3)	–
		In specialisation (3)	Registered nurse (1)	

**Fig. 1:**
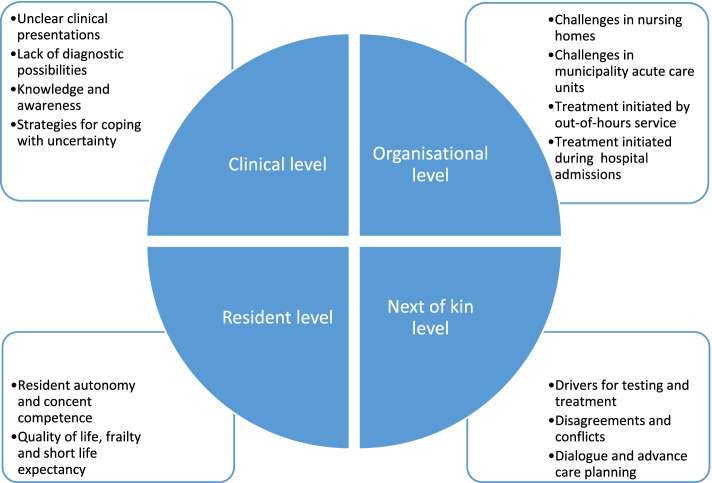
Overarching levels (in the circle) and associated themes affecting antibiotic prescribing by physicians nursing homes

We identified thirteen themes grouped into four main overarching levels affecting antibiotic use during the analysis, ranging from individual to external and systemic factors (Fig. 1). Most of the barriers and facilitators described by the informants applied to both NHs and MACUs. We have chosen to use the term NH further in the article when discussing factors that apply to both types of institutions, while we specify when factors were applicable only to NHs or MACUs.

### Barriers and facilitators at the clinical level

#### Unclear clinical presentation

Unclear clinical manifestations caused by infections, especially in cognitively impaired residents, were regarded as major contributors to diagnostic uncertainty and difficult treatment decisions. Some physicians highlighted the difficulty of distinguishing viral from bacterial respiratory tract infections, due to a perception that frail and old residents with viral respiratory tract infections often present with typical hallmarks of bacterial infections like fever, crackles over the lungs and increased C-reactive protein concentrations. Identifying bacterial aetiology was pointed out as a relevant challenge, especially during flu seasons, which often led to uncertainty among physicians regarding the initiation of antibiotics. In such cases, the resident’s general condition was described as decisive for whether antibiotic treatment should be initiated. Several physicians described that they had a lower threshold for starting antibiotic treatment in residents with a chronic obstructive pulmonary disease when in doubt about the microbiological cause of the respiratory infection. Diagnosis and treatment of urinary tract infection (UTI) was perceived as challenging, especially in demented residents, due to non-specific symptoms, poor anamnesis and high prevalence of asymptomatic bacteriuria, leading to a high level of uncertainty. Both physicians and nurses described several approaches to uncertain UTI situations, including watchful waiting, intravenous fluid therapy and antibiotic prescribing to be on the safe side. With uncertain focus of infections, several of the physicians and nurses described that broad-spectrum antibiotics were often prescribed to cover both the airways and urinary tract system. One of the physicians claimed that in cases where residents presented with new-onset non-specific symptoms, like confusion and agitation, it was better to try a short-term course of antibiotics than more side-effect burdened medications.

*Physician, male, 35 - 39 years: “They do not have the clear urinary tract infection symptoms. They can be agitated and have a positive urine stick. Instead of starting up with haloperidol or something similar, it is after all a bit better to give a course of pivmecillinam to check if a urinary tract infection is the cause. Therefore, you treat a little more on vague indications.”*

#### Lack of diagnostic possibilities

Lack of diagnostic possibilities was described as a general barrier for both the diagnostic process and for the choice of antibiotic by both professions. On-site x-ray was an opportunity they missed, mainly when dealing with emerging cases of respiratory infections to avoid unwanted and burdensome referrals to the local hospital. One physician described a case that involved a resident who had experienced several respiratory infections, leading to multiple antibiotic treatments. After referring the resident to an x-ray investigation at the hospital, lung cancer was identified. The informant emphasized that the resident could have been spared from many antibiotic courses if inhabiting an on-site x-ray at the NH. The lack of diagnostic possibilities were also described as a barrier for narrow-spectrum antibiotics, as it often led to a “better safe than sorry” approach.

*Physician, male, 35 – 39 years: “We are first-line service, so we do not have all the diagnostic tools. When you get a case with no clear clinical focus, and you have not performed a good advance care planning, I feel a bit trapped. I do not want to refer the patient to the hospital, but still you want to feel that what we do is justifiable. Then it happens that you end up with broad spectrum (antibiotics), and it is often with a bit of distaste, right?!”*

Among the nurses, a primary concern was an often prolonged time from sampling to blood biochemistry and bacteriological cultivation results. This was regarded as a barrier to treating infections in accordance with the National guidelines for antibiotic use and, potentially, leading to decisions not to prescribe the recommended first-line antibiotics.

#### Knowledge and awareness

The physicians and nurses pointed to a shortcoming of knowledge, mainly amongst auxiliary nurses and registered nurses, regarding indication, sampling and interpretation of point-of-care test results as a persistent and major barrier to appropriate antibiotic treatment of residents. Not interpreting the test results alongside the clinical signs and symptoms was described as a recurrent problem in the diagnostic process.

*Physician, male, 40 – 44 years:* “*The next day the C-reactive protein has risen, but the fever has gone down and the patient is out of bed. Then there are some who think that the antibiotics does not work … and then... then you have to quickly sign up for a course and start paying attention.”*

Both professions emphasized increased knowledge regarding the correct use of diagnostic tests as one of the most important measures of the intervention. In particular, the indication and interpretation of urine dipstick and C-reactive protein tests were mentioned.

To increase awareness among healthcare professionals, concerning own antibiotic prescribing practices and the development of antimicrobial resistance (locally and globally) was described as an additional intervention measure to facilitate prudent antibiotic use.

*Physician, female, 40 – 44 years: I gave the introductory antimicrobial resistance lecture, from the first conference meeting, to my nurses. When they saw the maps changing color throughout Europe, from mainly green to mainly red countries, there was a gasp from the assembly. Therefore, I think with good and correct knowledge it at least makes it easier and safer for me to explain and justify my choices to my colleagues.*

#### Strategies for coping with uncertainty

The physicians and nurses mentioned several strategies to counteract diagnostic uncertainty and thereby facilitate appropriate antibiotic use. Watchful waiting, often combined with intravenous fluid treatment, was described as a commonly used strategy, especially when dealing with suspected but uncertain UTI cases. Utilization of a urine culture to avoid unnecessary antibiotic treatment was additionally lifted as a strategy when encountering non-specific UTI symptoms.

*Physician, male, 35 - 39 years: “When speaking of UTIs, which is where perhaps the most disagreement is, I think if I am in doubt "yes, we’ll send it for culture". It takes a few days or a week until the culture is ready, and by that time the resident has become better or has developed more clear symptoms. Then I gain some time on it, and can postpone or avoid antibiotics.”*

Some nurses and physicians described the clinical checklist for suspected UTIs, offered to the participating institutions as part of the intervention, as a valuable and effective tool in reducing the number of antibiotic treatments in pending cases of UTIs. One perceived reason for this was that the threshold for sampling urinary dipstick tests increased amongst both auxiliary and registered nurses, leading to fewer tests being presented to the physician for evaluation. Finally, a referral system to the local hospital for diagnosis, treatment decision and level of care, informally called a “diagnostic loop referral”, was highlighted as an additional strategy when dealing with diagnostic uncertainty. This system was mainly utilized by the informants working at MACUs, and to some extent by the NH informants.

### Barriers and facilitators at the resident level

One of the physicians described the balancing act of antibiotic treatment in NH residents, and hence life-prolonging treatment, as operating in “gray areas”.

#### Resident autonomy and consent competence

Most of the nurses and physicians described an increased focus on assessing consent competence in NHs in recent years. In addition, several of the physicians and nurses emphasized the importance of the patients’ voice regarding antibiotic treatment, even if cognitively impaired and apparently consent incompetent.

*Nurse, female, 35 – 39 years: “We have demented residents who say: "No, I am now so ill that this is not compatible with life". And it's a bit like, what do you express in your own illness when you are there? We have demented pneumonia patients who can answer us: "Yes, thank you. It's nice that the doctor has been here, but I will not take any pills". Then you have those who want treatment for all it is worth.”*

Both professions expressed that if a consent-competent resident, and to some extent consent-incompetent residents, express a specific wish not to be treated with antibiotics in case of a life-threatening infection, the residents wish was usually respected. Nevertheless, some nurses described that it was not unusual that physicians still initiated antibiotic treatments against the residents wish not to receive treatment. Several factors were perceived as barriers to good judgment and decisions regarding antibiotic treatment in end-of-life situations. These included poor advance care planning, lack of residents own voice, cognitive impairment, and residents changing their minds regarding antibiotic treatment during an ongoing infection. Nevertheless, according to both nurses and physicians, such cases often culminated in antibiotic treatments.

*Physician, male, 35 – 39 years: “Then you have that “fresh product” as you mentioned earlier, where you have a patient who earlier said it does not want any life-prolonging treatment. Then the patient gets an infection and then they want treatment, and then they get (antibiotic) treatment.”*

#### Quality of life, frailty and short life expectancy

One decisive factor that was perceived as crucial regarding life prolonging treatment or not, including antibiotic treatment, was residents’ quality of life. Several different factors influencing quality of life were mentioned, such as familiar joys, a wish to experience important events (the next Olympics, a wedding, etc.), or simply to enjoy good food. When evaluating the quality of life, the physicians and nurses expressed that this was an assessment preferably done by residents themselves. In cases with an absent resident voice, both professions described that they usually involved next of kin to elicit information regarding the resident’s earlier preferences. If the involvement of the next of kin did not provide adequate information, the choice of treatment was to be decided by the responsible physician. One physician described that degree of cognitive impairment, pain and agitation were the most essential prerequisites for assessing the quality of life in residents unable to communicate themselves. There was a a general agreement among both nurses and physicians throughout the interviews that antibiotic treatment at the expense of residents’ quality of life was considered unethical and inappropriate.

*Physician, male, 35 – 39 years: “If the measure you take to live longer takes away the quality of life....”*

*Physician, male, 40 – 44 years: “Then it may not be the right measure.”*

One physician shared that many antibiotic treatment courses in NHs are both medically and ethically inappropriate, as it often prolongs residents’ suffering. According to the same physician, the reason for this, and hence being a barrier to proper antibiotic treatment in these situations, was that physicians refuse to decide to refrain from treatment as it is perceived as unpleasant. Furthermore, that the prescriber should assess the level of frailty and underlying disorders before initiating life-prolonging antibiotic therapy and reflect on the life situation the resident eventually would return to, given a successful treatment. Achieving this, according to the same physician, would facilitate both the medically and ethically appropriateness of antibiotic therapy in NH residents. The same physician also applied this to residents experiencing recurring bacterial infections and in cases where the preferred antibiotics lacked effect, leaving the question of whether to change to broader spectrum antibiotics or not.

*Physician, male, 40 – 44 years: “You have the option to start with penicillin, then it does not work so you add gentamycin, and if that does not work you switch to cefotaxim. If you choose that road, it is clearly not the right way to go with someone that frail in the first place. Although you might do it correct medically, you are ethically completely out of your mind.”*

Antibiotic treatment of palliative and pre-terminal residents with short life expectancies generated mixed responses. Several physicians expressed a tendency to treat palliative care residents with antibiotics primarily with a symptom-based, not life-prolonging, approach to relieving pain and discomfort associated with the infection. On the other hand, the majority of both nurses and physicians described a clear reluctance towards treating pre-terminal patients with antibiotics, including treatment to relieve symptoms.

*Physician, female, 35 – 39 years: “It seems directly unethical really. If you think they will die in a short time, giving antibiotics, I do not know. There are also side effects. I cannot see many situations where it can be justified.”*

### Barriers and facilitators at the next of kin level

#### Drivers for testing and treatment

Despite health personnel considering the treatment medically and ethically inappropriate, influence and pressure for antibiotic treatment from residents’ next of kin were described as a persistent barrier towards correct antibiotic use in NHs. Both the physicians and nurses expressed that diagnostic and antibiotic treatment should be based on residents’ wishes and medical decisions made by physicians. However, several informants from both professions were inclined to give in to non-coherent wishes and pressure from the next of kin. Lack of residents’ own voice, and cases where residents’ earlier wishes prior to the incapacity were unknown, were described as potential conflict-generating situations. Some nurses even described cases where residents clearly had expressed specific treatment wishes, but through persuasion by next of kin had chosen to change their position regarding treatment per the relatives’ wishes.

*Nurse, female, 60 – 62 years: “This man, at that time, he said clearly and unequivocally: "it is I who decide". Then the relatives persuaded him when we were not present. Therefore, I think there is a lot of hassle with family members.”*

#### Disagreements and conflicts

Disagreement between health care professionals and next of kin was widely discussed in several interviews as a common challenge concerning antibiotic treatment. Regarding frail elderly residents, the nurses and physicians described relatives who exhibit unrealistic expectations of the treatment effect of antibiotics and do not understand why diagnostic testing needs a clinical indication. Fear of negative coverage in the local newspaper or filing complaints to the municipality’s management department were reasons described for giving in to pressure from next of kin. Another reason for succumbing to the wishes of residents’ next of kin was the experience that this approach generated less work for the physician, as dealing with disagreements and conflicts were time-consuming and tiring. When exposed to strong disagreements regarding antibiotic treatment, some expressed that they simply chose to follow the wishes of next of kin to avoid conflicts. One physician explained that in cases where family members demanded antibiotic treatment in apparent unethical or medically futile situations, he often decided to treat with a narrow-spectrum antibiotic knowing that it would have no effect at all on the infection. Others described compromise-based approaches, which resulted in an antibiotic treatment attempt of two or three days, with subsequent termination of the treatment if the resident did not show signs of improvement.

*Nurse, female, 60 – 64 years: “But the grandson of the resident, who himself was a doctor, would not give up. Therefore, we talked with our physician and expressed that this was not correct. Then we decided to prescribe antibiotics for two or three days and then discontinue the treatment.”*

Some of the nurses further expressed an impression that residents’ relatives over the years increasingly have gained power concerning diagnostic procedures and initiating antibiotic treatment.

*Nurse, female, 45-49 years: “Whom are we actually treating? Are we treating the resident or the relatives?”*

#### Dialogue and advance care planning

Early stage dialogue with residents’ next of kin, often connected with advance care planning, was highlighted as facilitators for achieving ethically and medically correct antibiotic treatment of NH residents. Perceived benefits of early dialogue included building trust relationships, maturing and curbing relatives for future deteriorations in health and deciding level of antibiotic treatment prior to these events. Professional experience of healthcare workers, continuity in terms of full-time employment of physicians, and collaboration and agreement between health care professionals on complex issues regarding antibiotic treatment were described as important facilitators for trust building with next of kin. Although next of kin was generally described as needing repeated reality-oriented conversations concerning conflicting issues, such dialogues were considered an essential measure for ethically and medically correct antibiotic treatment.

*Nurse, female, 60 – 64 years: “The patient has a serious illness not yet diagnosed, which the hospital has chosen not to investigate any further. Therefore, if we do not talk openly about this the relatives might wonder why in the world we do not treat their dad, right?! Such cases need clarification.”*

Advance care plans, applying primarily for long-term-care NHs, were generally seen as valuable, reassuring and important by both physicians and nurses when dealing with new-onset infectious conditions, mainly concerning whether the resident should receive antibiotic treatment or not.

### Barriers and facilitators at the organisational level

#### Barriers and facilitators in nursing homes

Deficient staffing resources, especially concerning physicians, were described as an important barrier in terms of optimizing diagnostics and antibiotic treatment of residents. By having frequent access to the permanent NH physician, who inhabits knowledge of the residents and their medical history, this was perceived as a benefit for the residents themselves and facilitate both medically and ethically appropriate use of antibiotics.

*Physician, male, 40 – 44 years: “By being present every day, I think you get to use antibiotics in a much better way compared to arriving at a NH to attend an ill resident you do not know from before. Then it is much easier to think, “yes we’ll start antibiotic treatment because he is ill.”*

Both professions described the collaboration between nurses and physicians as non-problematic in terms of antibiotic treatment. Some physicians emphasized that due to the intervention, the collaboration worked better because the nurses to a lesser extent conducted point-of-care testing on their own initiative. Likewise, some physicians also highlighted decreased pressure from the nurses to initiate antibiotic treatment during and after the intervention, further adding to better collaboration between the two professions. Both professions pointed to the crucial role of nurses regarding the diagnostic process, where several of the physicians expressed that the nurses literally acted as their “eyes and ears” in many clinical decisions.

*Physician, female, 40 – 44 years: “We are very dependent on the nurses, it is therefore very important that they have a competent clinical view and that the collaboration works well.”*

Similarly, the nurses perceived their role in clinical decision-making processes as significant, and thus having a major impact on physicians’ clinical decisions. One physician pointed out that if a nurse wanted a resident treated with antibiotics, the nurse would have no problem convincing the physician into treating the resident.

*Physician, male, 35 - 39 years: “Yes, so it is how they (the nurses) describe it. They will get a cure for urinary tract infection if they want. They can report that the patient is more restless, has frequent urination and so on.”*

Some physicians and nurses described two potential barriers of appropriate antibiotic use regarding the nurse role. First, different nurses may have consistently different interpretations and reports of clinical observations. Secondly, nurses’ accuracy in relation to adherence regarding dosing intervals of oral antibiotics was described as often inaccurate, while with intravenous antibiotics the adherence to the intervals was usually accurate. Some of the nurses confirmed this, and described that one possible reason may be that residents treated with oral antibiotics are not considered as ill as those receiving intravenous antibiotics.

#### Challenges specific for municipal acute care units

One issue that applied explicitly to MACUs was that the diagnosis given in referral letters from general practitioners (GPs) and emergency physicians (OOHS) often was perceived as deliberately incorrect. Several of the MACU nurses and one MACU physician expressed a suspicion that the referring physicians often used wrong referral diagnosis as a shell hide for the real reason for admittance. UTIs and dehydration were mentioned as frequently used diagnoses to justify admissions to MACUs, while the real reasons for referral often were perceived as pressure from the patient’s relatives or the home nurse service regarding inclining difficulties living at home due to age, cognitive impairment and frailty. Referrals to MACUs often include an antibiotic initiation plan when the admission diagnosis is an infection and is usually not re-evaluated until the MACU physician returns to the ward. Several of the MACU nurses looked upon this as a barrier of correct antibiotic treatment, as many of the referred UTI cases were actual cases of asymptomatic bacteriuria not requiring antibiotic treatment.

*Nurse, female, 25 – 29 years: “We get patients referred with a plan, like an antibiotic regimen. But the real reason for admission is that relatives are going on holiday … they take a urine sample, find something on the dip stick and then they are admitted to us.”*

#### Treatment initiated by out-of-hours service

The nurses generally expressed that OOHS was something they strived to avoid using as far as possible, as contacting the OOHS was tantamount to calling in a treatment order.

*Nurse, female, 35 – 39 years: “You call in an order, and you get what you ask for.”*

Regarding antibiotic treatment, several of the nurses and physicians in the interviews shared the opinion that OOHS physicians had a lower threshold for initiating broad-spectrum antibiotics. Lack of knowledge concerning resident history and lack of clinical examination of residents as the treatment decision often happens by telephone consultation were described as main barriers towards appropriate antibiotic use when initiated by the OOHSs.

*Physician, male, 30 – 39 years: “I think that the OOHS physicians do not know the residents very well, and to them an ill resident is an ill resident. They do not think about what kind of quality of life this patient already has. So they are faster to start treatment, and perhaps more broad-spectrum and more intravenous (treatment) than we might have done. Because they do not see the whole picture.”*

To avoid involving the OOHSs, some physicians pointed out they had agreements with their NHs to be available on phone outside working hours while others did not follow such practice as it resulted in too many inquiries after hours.

### Treatment initiated during hospital admissions

Several of the physicians and nurses shared thoughts and frustration regarding overtreatment of NH residents initiated during hospital stays. Palliative care and other residents with short life expectancy and reduced quality of life returning to the NHs with ongoing antibiotic treatment that clearly would outlive the resident itself, were perceived as particular problematic cases.

*Physician, female, 50-54 years*: *“When is enough, enough? One resident returned from the hospital with a gallbladder infection with intravenous antibiotics and nutrition, but the resident was over ninety years old with severe heart failure. Then you feel trapped with how long are you going to hold on, when are you supposed to stop the treatment? I felt the hospital over-treated the resident.”*

In general, the physicians described that they seldom challenged or re-evaluated hospital-initiated treatment, even in cases where antibiotic treatment clearly was questionable. Only if antibiotic treatments were excess broad-spectrum, further degraded residents’ quality of life or were obviously medically futile, some physicians expressed that they might contact hospital colleagues to discuss the treatment. Perceived barriers to challenging hospital-initiated treatments included respecting hospital physicians being specialists, better diagnostic possibilities at the hospital and difficulties defending change of treatment towards residents’ next of kin.

*Physician, female, 40 – 44 years: “Yes, so I know a little about a lot, they know a lot about less. It is natural that they should be better than me at this I think. If I am very stunned, I call to ask of course. However, to change (the treatment)? Then it has to be completely outrageous.”*

## Discussion

We identified four overarching levels covering thirteen themes affecting the appropriateness of antibiotic use in primary care institutions: Barriers and facilitators 1) at the clinical level, 2) at the resident level, 3) at the next of kin level, and 4) at the organisational level (Fig. 1). Our main finding was the unclear clinical presentation of symptoms and lack of diagnostic possibilities as persistent barriers of appropriate antibiotic use after the quality improvement programme. Increased knowledge and awareness, appropriate use of point-of-care tests, increased availability of the permanent NH physicians and early and frequent dialogue with the residents’ next of kin were important facilitators of appropriate antibiotic use.

Corresponding well with a previous Dutch study [16], we found that unclear clinical presentations greatly contribute to diagnostic uncertainty. Correct diagnosis of infections with an emphasis on distinguishing asymptomatic bacteriuria (ABU) from cystitis, was a major educational focus of the intervention. Although the informants described an improvement regarding these issues after the intervention, they still expressed a clinical reality where unclear clinical symptom presentations played a significant role as a barrier towards medically appropriate antibiotic use. In line with a previous NH interview study [33], the informants expressed that non-specific functional and behavioral changes often were wavered in the clinical assessment of suspected UTI cases. When suspecting a UTI, both based on specific and non-specific UTI symptom presentation, a further examination by urine dipstick analysis and urinary culture is standard practice. Taking into account the findings of Sundvall et al. [44] that positive urine cultures were as common in NH residents with as without non-specific symptoms, the continued need for education on correct clinical assessment of UTIs in NH residents must be emphasized. Several informants highlighted the clinical UTI checklist on observed signs and symptoms as an effective facilitator for increasing the threshold for a sampling of urinary dipstick tests. Especially the nurses valued the checklist and described an observed decrease in the number of sampled urinary tests and antibiotic use for suspected UTIs after the checklist implementation. Two recent studies utilising checklists for signs and symptoms of suspected UTIs in NH residents reported improvements in the use of UTI antibiotics [45, 46]. Based on the findings in these studies and our study, clinical checklists as diagnostic guiding tools appear to be an effective and easy to implement measure facilitating appropriate antibiotic use in NH residents.

Lack of on-site diagnostic tools and resources was perceived as a persistent barrier in achieving medically optimal antibiotic treatment and has previously been described [16, 34]. The nurses expressed frustration of the delay in obtaining laboratory test results, especially from urine cultures. The physicians did not mention this particular issue as a major clinical challenge to the same extent. One reason may be that the National Guidelines for antibiotic use in NHs recommend three empirical first-line antibiotics for UTIs, providing the physicians with different antibiotic choices in case of treatment failure [47]. The description of utilizing urine cultures as a facilitator to buy time and thereby avoid immediate antibiotic initiation when exposed to uncertain UTI cases is to our knowledge not described before. Alongside increased knowledge regarding clinical and laboratory test evaluation, the informants emphasized increased awareness concerning their own antibiotic use through workshops as an important facilitator in achieving medically appropriate antibiotic use. This academic detailing approach has previously been shown to facilitate reduction and appropriateness of antibiotic use in both general practice and NHs [46, 48]. We, therefore, encourage such an approach when planning future NH antibiotic stewardship programs.

Awareness and emphasis on patient autonomy and consent-competence were described as important facilitators for ethically and medically appropriate antibiotic prescribing. The informants shared the opinion that consent-competent residents, able to express treatment desires, should be the major guiding factor in treatment decision-making. This finding somewhat contradicts the findings of Klomstad *et.al*. where the patients’ individual preferences seemed to have a more peripheral role in the decision-making regarding life-prolonging treatment [49]. In contrast, lack of residents ability to express themselves, due to hearing or speech difficulties, worsened general condition and cognitive impairment, and residents’ ambivalence regarding treatment, were described as persistent barriers to appropriate antibiotic use. When one or more of these factors are present, one consequence may be that adequate anamnesis is made more difficult, in turn leading to difficulties and uncertainty regarding correct medical diagnostics and antibiotic treatment. Another possible consequence may be that the patient’s desire for treatment remains unknown to the responsible physician, which increases the possibility for initiating ethically debatable antibiotic treatment. These barriers are not easily solved and rest mainly on individual assessments by health care professionals responsible for the treatment. Nevertheless, we believe that these barriers can be improved by increasing the clinical knowledge regarding infection diagnostics and thus promoting confidence when exposed to unclear and demanding situations. Regular colleague forums and the opportunity to confer with other colleagues on-site or via telephone would most likely strengthen decision-making in similar cases. In addition, advance care planning, covering antibiotic treatment clarification, was described as a key facilitator for appropriate life-prolonging treatment when dealing with uncertainty generating resident factors. These findings correspond well with other studies reporting that advance care plans often are appreciated and has a central role in the decision-making process in NHs, including infection treatment [16, 50].

Antibiotic therapy in palliative medicine is an area permeated by ethical issues without a single correct answer. Although most informants shared the agreement that one should avoid antibiotic treatment in residents with obvious short life expectancies, some informants expressed willingness to treat palliative care residents with antibiotics to relieve discomfort associated with infections. This tendency corresponds well with the findings of a recent North American descriptive survey, where most participating NHs reported that end-of-life residents likely would receive antibiotics if UTI was suspected [51]. Therefore, future antibiotic stewardship programs should address these issues in an attempt to make NH and MACU physicians better prepared in such situations. The main message of such an approach should always be to consider restraining from antibiotic treatment if the residents’ quality of life most likely will be worsened by the treatment, or if the antibiotic treatment most certainly will be medically futile.

The nurses frequently described next of kin’s expectations as one of the most considerable barriers towards achieving medically and ethically appropriate antibiotic treatment. The decision-making influence from next of kin is well known from previous NH studies [52, 53]. Antibiotic treatment in such cases often conflicts with good clinical practice, highlighting the need for better interaction and information exchange towards residents’ next of kin. Giving in to pressure as it is less time-consuming, and fear of complaints and unpleasant media coverage are previously described reasons for giving in to pressure from next of kin [52, 53]. Advance care plans, including early-stage and repeated dialogue with next of kin, were regarded as facilitators for avoiding disagreement and conflicts. Based on a previous Norwegian NH study, there is further room for improvement by increasing the proportion of conducted advance care plans in NH residents [49]. Focus on readily available and clear advance care plans familiar to the NH healthcare professionals, should therefore be a priority in future antibiotic stewardship programs.

However, situations presenting contradictions between treatment decisions and one’s own work ethic and known good clinical practice may not be mitigated by advance care plans, dialogue with next of kin, increased clinical knowledge and collegial conferring alone. Increasing the professional knowledge and experience of care givers in the field of ethical issues in NHs through education, guidelines and ethical reflection groups, may contribute to health personnel becoming more robust in the face of such challenging situations. During the opening conference of the intervention, a professional presentation alongside a workshop covering ethical aspects of antibiotic treatment in NH residents were held in this regard. Although not mentioned by the informants during the interviews, in demanding ethical cases where the above components fall short as to solve the issue, a clinical ethics committee may be contacted for advice and guidance in specific cases. All major health trusts in Norway and some municipalities have a clinical ethics committee which may be contacted by NHs when needed.

Lack of permanent physicians and infrequent regular medical visits is a common everyday situation, especially for small NHs in Norway [18] and abroad [54]. The informants in our study highlighted the increase of these two factors as key in facilitating optimal use of antibiotics, as it would lead to a better knowledge of residents’ medical history and settled advance care plans. In addition, this would further reduce the involvement of OOHSs, which by the informants would reduce the likelihood of unnecessary and broad-spectrum antibiotic prescribing.

Our findings regarding the influence of nurses in the diagnosis and treatment of infections are by no means unique [16, 33, 34]. Given the amount of time nurses interact with residents compared to physicians, it is natural that physicians trust and emphasize the reports from this occupational group, highlighting the importance of adequate and sound clinical observations and evaluations from the nurses. However, the large variation in the quality of observations and reporting from different nurses is worrying, potentially leading to both over- and under-prescribing of antibiotics. Furthermore, the descriptions about the inaccuracies of the nurses regarding oral antibiotic dosing intervals can, in a worst-case scenario, lead to inadequate effect of antibiotic regimens. These barriers demonstrate that antibiotic stewardship in NHs, to be as effective as possible, should include nurses on an equal footing with physicians.

MACUs are a relatively new service in the Norwegian health service, and research in the field is so far scarce. In the current study, the informants expressed a suspicion that several of the referral diagnoses stated by GPs and OOHS physicians are used as cover for other conditions or situations less suitable for admittance to MACU wards. By exploiting the large incidence of asymptomatic bacteriuria in the elderly as a gateway to MACUs, this increases the risk of unnecessary antibiotic prescribing. Cumbersome and defiant collaboration between MACU employees and GPs regarding admittance to MACU’s, as well as vague admission criteria as perceived by GPs, have previously been described by Johannessen *et.al* [55]. Together with our findings, this further strengthens the need for better collaboration between the various primary health care services and more explicit admission criteria to MACUs to achieve the best possible use of antibiotics.

Previous Norwegian studies have shown that hospital-initiated antibiotic treatment in some instances should be challenged, including the spectrum of the initiated antibiotic treatment and the outlined treatment duration [56, 57]. Despite addressing this issue during the intervention meetings, where the participants were encouraged to evaluate critically, and if indicated challenge hospital initiated antibiotic regimens, the informants still described a reality lacking such an initiative. The barriers described as driving this reluctance; feeling of being less of a specialist and differences in diagnostic opportunities, may be improved by increasing the clinical and theoretical knowledge and competence in NH physicians as well as to facilitate for easier conferring between NH- and hospital physicians. Together with the findings of a previous Norwegian study*,* which describe communication failure at all stages of the patient pathway in the collaboration between NHs and hospitals [58], this area calls for further focus in future antibiotic stewardship programs.

### Strengths and limitations

The main strength of the study is the investigation of not only physicians’ perspectives, but also perspectives of NH nurses given their obviously significant role in the antibiotic decision-making. Furthermore, the informants’ wide range in age and working experience resulted in rich and varied feedback that broadly and realistically embraces the everyday clinical life in Norwegian primary care institutions.

As a limitation applicable to most qualitative research, this study cannot firmly conclude to what extent each identified barrier and facilitator affects antibiotic prescribing in NHs and MACUs. In order to present precise assumptions around the magnitude of each factor, future observational and quantitative studies are warranted. Another possible limitation of the study may be the composition of informants in the first four interviews, in which both physicians and nurses participated together. This may have resulted in some informants being reluctant to express themselves credibly and truthfully about their own role and concerning the other occupational group present during the interviews. Based on this potential limitation, we conducted the two last interviews with only physicians present in one and only nurses in the other one, without observing any apparent differences in the feedback or dynamics compared to the first four interviews. We therefore believe that if such impact has found place during the mixed interviews, it has been of minor relevance to the results of the study. There might have appeared changes in the investigated field in 4 years’ time between data collection and publication of the results. However, we have not identified any new relevant guidelines or published literature from Norwegian NHs addressing the area of interest in the time period between data collection and publication. We, therefore, believe our results and conclusions stands viable and firmly and adds valuable knowledge to a field where prior research is scarce. Lastly, when interpreting the results of the study, it is important to remember that our findings are based on descriptions and perceptions of the physicians and nurses, and thus lacking the views and experiences of residents, next of kin, other health care professionals from other health services and the management representatives from the institutions.

## Conclusions

After the completion of a one-year antibiotic quality improvement intervention, our focus group study reveals a wide variety of persistent barriers influencing antibiotic prescribing in the participating NHs and MACUs. Unclear clinical presentation of symptoms, lack of diagnostic possibilities and pressure from next of kin were perceived as major barriers to appropriate antibiotic use. On the other hand, increased knowledge and awareness, appropriate use of point-of-care tests, increased availability of permanent NH physicians and early and frequent dialogue with the residents’ next of kin were important facilitators of appropriate antibiotic use. The influence of nurses in the decision-making process was by both professions described as profound. We encourage targeting these factors in future antibiotic stewardship programs to achieve the most adequate antibiotic treatment possible. Future studies should lean towards quantitative and observational methods to gain more knowledge of how to overcome barriers and contribute to practice- and implementation developments to ensure optimal antibiotic prescribing to elderly patients.

## Data Availability

The datasets used and/or analysed during the current study are available from the corresponding author on reasonable request.

## Abbreviations

NH: Nursing home
MACU: Municipal acute care unit
UTI: Urinary tract infection
OOHS: Out-of-hours services
GP: General practitioner
